# Physical Activity and Healthy Aging: Functional, Cognitive, and Sleep Predictors Associated with Fall Risk in Older Adults

**DOI:** 10.3390/ijerph23070878

**Published:** 2026-07-06

**Authors:** Marilia Salete Tavares, Sara Lucia Silveira de Menezes, Walace Monteiro, Camila Tavares Rodrigues, Daniel Joppert, Thiago Rodrigues Gonçalves, Joana da Costa Pinto D’Avila, Paulo Henrique de Moura, Jorge Ferreira da Silva Junior, Adalgiza Mafra Moreno

**Affiliations:** 1 Exercise Physiology Laboratory, Postgraduate Program in Physical Activity Sciences, Universidade Salgado de Oliveira, Niterói 24030-060, Brazil; menezes@hucff.ufrj.br (S.L.S.d.M.); wallace.monteiro@nt.universo.edu.br (W.M.);; 2 Health and Aging Research Group, Universidade Iguaçu, Nova Iguaçu 26275-580, Braziljoana.avila@campus1.unig.br (J.d.C.P.D.); 0142076@professor.unig.edu.br (P.H.d.M.);; 3 Graduate Program in Education, Contemporary Contexts and Popular Demands (PPGEduc), Federal Rural University of Rio de Janeiro (UFRRJ), Seropédica 23890-000, Brazil; camila.51391953@prof.educa.rj.gov.br

**Keywords:** accidental falls, aged, exercise, sleep quality, cognition

## Abstract

**Highlights:**

**Public health relevance—How does this work relate to a public health issue?**

- Falls among older adults represent an important public health problem, associated with functional decline, hospitalizations, disability, and increased mortality.
- Sleep disturbances and cognitive impairment, combined with polypharmacy, emerge as relevant non-motor factors associated with fall risk in community-dwelling older adults.

**Public health significance—Why is this work of significance to public health?**

- The study shows that sedentary older adults tend to present poorer balance, reduced mobility, worse sleep quality (PSQI), and lower cognitive performance (MMSE), factors associated with a higher occurrence of falls.
- The findings reinforce the need for integrated approaches to fall risk assessment, including functional, cognitive, and sleep-related dimensions.

**Public health implications—What are the key implications or messages for practitioners, policy makers and/or researchers in public health?**

- Public physical activity programs may act as a protective strategy by improving balance, mobility, sleep quality, and cognition, contributing to fall reduction among older adults.
- Public health policies and practices should incorporate systematic screening of sleep quality, cognitive status, and multiple medication use as part of preventive actions in primary care and community-based programs.

**Abstract:**

Falls among older adults are a major public health concern due to their association with functional decline and increased healthcare utilization. This study investigated factors associated with fall occurrence by comparing older adults enrolled in a community-based physical activity program with sedentary older adults. This observational cross-sectional study included 67 older adults: 35 participants enrolled in the Niterói 60 Up program (G60UP; 68 ± 4 years) and 32 sedentary older adults in a sedentary comparison group (SCG; 70 ± 7 years). Assessments included anthropometric measurements, medication use, Timed Up and Go (TUG), Tinetti Performance-Oriented Mobility Assessment, Mini-Mental State Examination (MMSE), Pittsburgh Sleep Quality Index (PSQI), and orthostatic testing. Compared with the SCG, G60UP participants reported fewer falls during the previous year (0.26 ± 0.51 vs. 0.78 ± 0.83; *p* = 0.005), higher Tinetti scores (24.51 ± 2.54 vs. 18.28 ± 5.94; *p* < 0.001), shorter TUG times (8.66 ± 1.63 vs. 13.00 ± 4.31; *p* < 0.001), higher MMSE scores, better sleep quality (lower PSQI scores), and lower blood pressure and abdominal adiposity indicators. In the total sample, fall occurrence was associated with lower Tinetti scores (ρ = −0.416; *p* < 0.001), longer TUG times (ρ = 0.321; *p* = 0.008), older age (ρ = 0.328; *p* = 0.007), higher Conicity Index (ρ = 0.281; *p* = 0.021), and poorer sleep quality (ρ = 0.243; *p* = 0.047). No variable remained independently associated with falls in the exploratory multivariable logistic regression model. Participants enrolled in the 60 Up program presented more favorable functional, cognitive, sleep-related, and health-related profiles than those in the SCG. Due to the cross-sectional design, these findings should be interpreted as associations rather than causal relationships.

## 1. Introduction

Population aging has accelerated worldwide and represents one of the main contemporary challenges for health systems, particularly in low- and middle-income countries. In 2024, global life expectancy at birth reached 73.3 years, representing an increase of more than eight years since 1995. In parallel, the number of people aged 60 years and older is expected to rise from 1.1 billion in 2023 to 1.4 billion by 2030 [1].

In Brazil, this demographic transition has occurred rapidly and unevenly, reflecting historical social inequalities and disparities in access to health services and adequate living conditions. Data from the Brazilian Institute of Geography and Statistics (IBGE) indicate that adults aged 60 years or older accounted for more than 15% of the Brazilian population in 2022, with a continuous upward trend projected for the coming decades [2]. According to the United Nations World Population Ageing 2023 report, this shift has increased the demand for preventive strategies and long-term care aimed at maintaining functional capacity and autonomy among older adults [3].

However, increased longevity is not necessarily accompanied by improvements in quality of life. A growing gap between life expectancy and years lived in good health has been described as the “health gap” [4]. Health outcomes in older age reflect cumulative exposures across the lifespan, including sedentary behavior, socioeconomic vulnerability, multimorbidity, and limited access to preventive healthcare, all of which may contribute to functional decline and increase fall risk [4,5,6,7].

Falls are among the most frequent and severe adverse events affecting older adults and represent a major public health concern worldwide. It is estimated that falls account for approximately 700,000 deaths annually, and around 30% of older adults experience at least one fall each year [5,8,9]. In Brazil, falls have also become increasingly prevalent. Between 2000 and 2019, approximately 15,000 older adults died due to falls, with higher rates among individuals aged 70 years or older [8,9].

Data from SISAP-Idoso indicate that mortality rates due to falls nearly tripled between 2000 and 2017 [10]. In 2023, the Brazilian Society of Orthopedic Trauma reported 106,000 hospital admissions and 45,000 outpatient visits related to falls in the Brazilian Unified Health System (SUS) [11]. 

Fall risk in older adults is multifactorial and includes intrinsic and extrinsic determinants. Functional impairments such as muscle weakness, balance deficits, and gait disturbances are among the most consistently reported factors [12,13,14].

Autonomic dysfunction, including orthostatic hypotension, is also common in aging and has been associated with an increased risk of falls due to transient cerebral hypoperfusion and postural instability [15]. In addition, cognitive decline may compromise motor control and attentional processing, increasing the likelihood of falls, particularly among individuals with dementia, in whom fall prevalence may reach 70% annually [15,16,17,18].

Other relevant factors include polypharmacy and sleep disturbances. The concomitant use of multiple medications, especially those acting on the central nervous system and the cardiovascular system, may cause adverse effects such as daytime sleepiness, dizziness, impaired balance, and orthostatic hypotension, thereby increasing instability and the risk of falls. Likewise, poor sleep quality may compromise alertness, reduce functional capacity, and impair motor control, increasing the likelihood of accidents during walking [19].

Anthropometric and metabolic factors may also contribute to mobility limitations and fall risk. Abdominal obesity is associated with cardiovascular risk and impaired functional capacity, and indices such as the Conicity Index, waist circumference, waist-to-height ratio, and body mass index are widely used to assess body fat distribution and related health risks [16,20,21].

Sedentary behavior is frequently observed among older adults and directly undermines functional independence, often creating a self-reinforcing cycle in which reduced mobility promotes further inactivity and accelerates motor decline. This process may contribute to increased adiposity, loss of muscle mass, and reduced ability to perform daily tasks, thereby increasing vulnerability to falls [21,22,23].

Furthermore, physical inactivity has been associated with psychological and clinical conditions such as low self-esteem, stress, depressive symptoms, chronic diseases, and reduced physical capacity, which may further increase fall susceptibility and reinforce sedentary patterns [22,24,25,26].

In contrast, regular physical activity improves balance, muscle strength, mobility, and functional capacity, supporting the maintenance of independence in older age [25]. In Brazil, national public policies have emphasized the promotion of active aging. The National Policy for Older Adults, established in 1994, supports strategies to improve quality of life and autonomy, and several public initiatives have been developed to encourage physical activity in this population [24,27,28].

Among these initiatives, the 60Up project, implemented in the city of Niterói, aims to promote physical and mental health through structured physical exercise, outdoor activities, and cultural practices [29,30].

Falls may result in fractures, loss of independence, disability, prolonged hospitalization, surgical interventions, and long-term rehabilitation, generating a substantial burden for healthcare systems, families, and society [14,31]. Although regular physical activity has consistently been associated with better functional capacity and lower fall occurrence, falls are recognized as a multifactorial geriatric syndrome for which isolated motor interventions may be insufficient. Increasing evidence indicates that non-motor factors, including polypharmacy, sleep disturbances, and cognitive impairment, interact with functional deficits and contribute to fall vulnerability [13,32,33,34,35].

Beyond impaired balance and reduced mobility, cognitive performance and sleep quality have emerged as important components of fall risk assessment. Cognitive impairment may affect executive function, attention, dual-task performance, and motor planning, thereby compromising gait stability and increasing fall susceptibility. Likewise, poor sleep quality has been associated with daytime sleepiness, impaired attention, slower reaction time, reduced postural control, and increased fall occurrence. Importantly, cognitive impairment and sleep disturbances frequently coexist in older adults and may interact synergistically, accelerating functional decline and increasing vulnerability to falls. Therefore, the combined assessment of cognitive performance, sleep quality, and functional mobility may provide a more comprehensive understanding of fall-related risk factors and support multidimensional prevention strategies for community-dwelling older adults [16,19,34,36,37,38,39]. 

Despite the growing body of literature on fall prevention and community-based physical activity programs, many previous studies have focused primarily on motor outcomes or have adopted qualitative approaches. Consequently, evidence integrating motor and non-motor factors—including cognition, sleep quality, anthropometric indicators, and cardiovascular health—remains limited. This multidimensional approach may improve the identification of older adults at greater risk of falls and contribute to the development of more comprehensive preventive strategies for healthy aging.

Therefore, the aim of this study was to compare functional, cognitive, sleep-related, anthropometric, and clinical characteristics between older adults enrolled in a public, free community-based physical activity program (G60UP) and a sedentary comparison group and to investigate the associations of these characteristics with self-reported fall occurrence during the previous 12 months.

## 2. Materials and Methods

### 2.1. Study Design

This was an observational, analytical, and cross-sectional study with a quantitative approach, conducted at the Exercise Physiology Laboratory of Salgado de Oliveira University, in Niterói, Rio de Janeiro, Brazil. Data collection and participant assessments took place between July 2023 and May 2025. All measurements were obtained from current participants enrolled in the Niterói 60 Up program and from sedentary older adults residing in the community, recruited during the same period.

### 2.2. Ethical Approval

The study was approved by the Research Ethics Committee (CAAE: 67496423.6.0000.8044). All participants received detailed information regarding the study objectives and procedures and signed an informed consent form prior to participation.

### 2.3. Participants and Sample Selection

A convenience, non-probabilistic sample of 67 community-dwelling older adults was included in this exploratory cross-sectional study. Participants were allocated into two groups according to physical activity status: the G60UP Group (*n* = 35) and the Sedentary Comparison Group (SCG; *n* = 32).

The G60UP Group consisted of older adults enrolled in the public physical exercise program “Niteroi 60Up” (30 women and 5 men).

The SCG included 32 older adults (25 women and 7 men) who reported no regular participation in structured physical exercise programs during the three months preceding data collection. All participants were apparently healthy and functionally independent. Participants were recruited by convenience from community settings in the municipality of Niteroi, Rio de Janeiro, Brazil. 

The primary objective was to generate preliminary evidence regarding potential associations between participation in a community-based exercise program, functional performance, sleep quality, cognition, and fall-related outcomes in community-dwelling older adults. 

**Figure 1:**
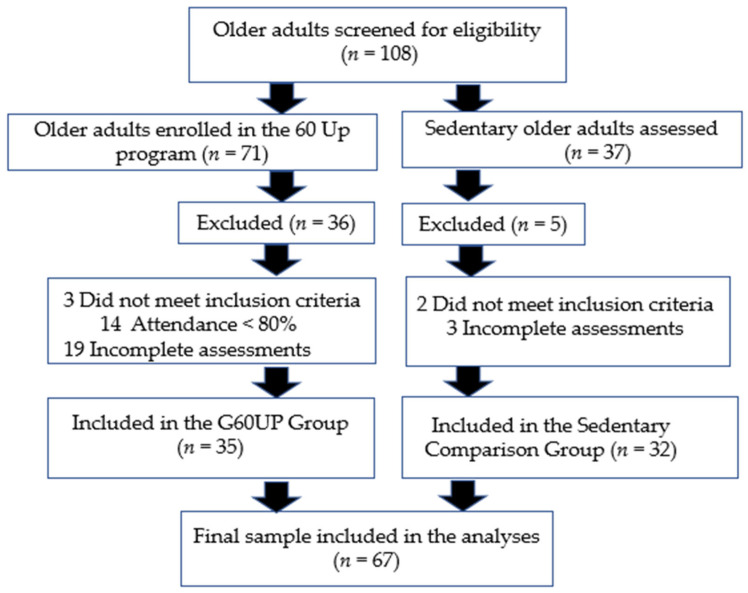
Flowchart of participant selection and inclusion in the study.

A total of 108 community-dwelling older adults were initially screened for eligibility, including 71 participants enrolled in the Niterói 60 Up program and 37 sedentary older adults. After the application of the inclusion and exclusion criteria and the exclusion of participants with incomplete assessments, 67 older adults were included in the final analyses, comprising 35 participants in the G60UP Group and 32 participants in the Sedentary Comparison Group (SCG). The participant selection process is summarized in Figure 1.

### 2.4. Exercise Program Description

The active group consisted of older adults enrolled in the Niterói 60 Up municipal physical activity program, a free public initiative designed to promote healthy aging, physical activity, and social participation among community-dwelling older adults. The program is funded and maintained by the Municipality of Niterói and coordinated by the Municipal Secretariat for Older Adults, Rio de Janeiro, Brazil. At the time of the study, it operated through 25 community-based centers distributed across different neighborhoods of the city and offered activities specifically tailored for older adults, including preventive gymnastics, ballroom dancing, social interaction groups, and choir activities.

For inclusion in the active group (G60UP), participants were required to have attended the gymnastics program for at least four consecutive months, with a minimum attendance rate of 80% during the period preceding data collection. To reduce variability in exercise exposure, only participants with a similar participation period (6–12 months) were included.

The gymnastics sessions were offered five times per week (Monday to Friday) and lasted approximately 60 min. The exercise protocol was structured to promote progressive cardiovascular and respiratory stimulation while maintaining participant safety. Sessions began with a warm-up phase consisting of approximately 5 min of deep breathing exercises, dynamic stretching, and stationary walking, performed at low-to-moderate intensity.

The main phase included functional and resistance-based exercises, such as squats using sticks and deadlift-like movements, performed for approximately 5 min each at moderate-to-high intensity. After a resting interval of approximately 10 min, participants performed rhythmic exercises accompanied by music, including simple dance steps and short choreographed sequences, also performed at moderate-to-high intensity.

The final phase consisted of static stretching and deep breathing exercises performed at low intensity to facilitate recovery and relaxation. Exercise intensity was classified as low (minimal physical effort, corresponding to warm-up and cool-down activities), moderate (increased heart rate and respiratory rate while maintaining the ability to converse), and moderate-to-high (marked cardiorespiratory demand with reduced ability to sustain conversation) [29,30].

All activities were supervised by qualified physical education professionals affiliated with the program. Safety procedures were standardized across centers, and program staff received first-aid training through a partnership with the local Fire Department. Individual exercise load, heart rate monitoring, and objective training volume were not systematically recorded. Therefore, exercise exposure was characterized based on attendance records and duration of participation rather than objective training-load measurements.

### 2.5. Inclusion and Exclusion Criteria

Individuals aged 60 years or older, literate, cognitively preserved, and able to ambulate independently were eligible. Participants were required to be physically able to perform gait and balance tests and to understand and follow the assessment instructions. For inclusion in the active group, participants were required to participate in the exercise program for at least four consecutive months. For inclusion in the sedentary group, participants were required not to have engaged in structured physical activity programs or regular exercise during the three months prior to the assessment.

Exclusion criteria included severe functional impairment, wheelchair dependence, or the use of walking assistive devices (e.g., cane or walker). Participants with severe visual or hearing impairments, neurological sequelae, relevant vestibular disorders, or any condition that could significantly compromise balance, mobility, or safe test execution were also excluded.

### 2.6. Assessment Procedures

After telephone screening, participants received standardized instructions regarding pre-assessment procedures and were advised to abstain from alcohol, caffeine, and physical exercise within 24 h prior to data collection. An investigative questionnaire (medical history form) was administered, including open-ended questions on personal data, health conditions, medication use, and history of falls.

After completion of the questionnaire, initial clinical assessments were performed, including measurements of vital signs (blood pressure and heart rate), anthropometric variables, and adiposity-related indicators. The assessed variables included body mass, height, body mass index (BMI), waist circumference (WC), waist-to-height ratio (WHtR), Conicity Index, and estimated visceral fat level obtained through bioelectrical impedance analysis (BIA).

Blood pressure was measured using an aneroid sphygmomanometer and a Rappaport stethoscope (Premium^®^, Ningbo, China). Heart rate and heart rate variability (HRV) were assessed using a Polar H10 heart rate sensor (Polar Electro Oy, Kempele, Finland). Height was measured using a wall-mounted stadiometer (Sanny^®^, São Bernardo do Campo, SP, Brazil) with a precision of 0.1 cm. Participants were assessed barefoot in an upright standing position, with their heels together, arms relaxed alongside the body, and the head positioned according to the Frankfurt plane, following standardized anthropometric procedures. Waist circumference was measured using a non-elastic measuring tape graduated in centimeters according to standardized protocols.

Body mass and estimated visceral fat level were obtained using the ITeknic IK-PCA001 smart scale (ITEKNIC Shenzhen Nearby Express, Shenzhen, China), which estimates body composition through bioelectrical impedance analysis. Participants were instructed to maintain a minimum fasting period of three hours, empty their bladder before the assessment, and avoid physical exercise during the previous 12 h. Measurements were performed with participants barefoot, wearing light clothing and no accessories, standing upright with feet parallel and slightly apart and arms extended alongside the body.

Body mass and estimated visceral fat level data were digitally recorded and stored in a cloud-based system for subsequent analysis [40]. Because assessments were conducted under field conditions, hydration status, recent food intake beyond the minimum fasting period, and medication use could not be fully standardized. Therefore, BIA-derived estimates should be interpreted with caution and not as laboratory-grade measurements.

BMI was calculated as body mass (kg) divided by height squared (m^2^). The WHtR was calculated as the ratio between waist circumference and height, both measured in centimeters. Values above 0.50 were considered indicative of increased abdominal adiposity and higher cardiometabolic risk [20].

The Conicity Index was calculated according to the method proposed by Valdez [41], using body mass, height, and waist circumference measurements. Body mass (kg) was divided by height (m), the square root of the resulting value was obtained, and the result was multiplied by the constant 0.109. Waist circumference (m) was then divided by this value to obtain the individual Conicity Index.

After the physical assessments, the Mini-Mental State Examination (MMSE) was administered to evaluate cognitive functions, including orientation, memory, attention, language, and visuospatial ability. The maximum score is 30 points, and education-adjusted cutoff points were adopted: <15 points for illiterate participants, ≤22 points for individuals with fewer than 11 years of education, and <27 points for those with more than 11 years of schooling [17,42].

Sleep quality was subsequently assessed using the Pittsburgh Sleep Quality Index (PSQI), a self-administered questionnaire consisting of 19 items that investigates sleep latency, duration, efficiency, and the use of sleep medication. The total score ranges from 0 to 21 points and was classified as good sleep quality when <5 points, moderate to poor sleep quality between 5 and 10 points, and severely impaired sleep quality when >10 points [43].

The orthostatic assessment was included as a complementary physiological evaluation aimed at investigating potential autonomic influences on fall-related vulnerability among older adults. Heart rate (HR) and heart rate variability (HRV) data were collected using a Polar H10 heart rate sensor (Polar Electro Oy, Kempele, Finland). RR intervals were recorded through a chest strap positioned in the precordial region and synchronized with the Polar Flow platform. The sensor was attached using an elastic chest strap, and the electrode areas were moistened with conductive gel to optimize signal acquisition [44,45,46].

All orthostatic assessments were conducted by the same research team, at the same location, and during the morning period to minimize environmental and circadian influences on cardiovascular responses. Participants remained at rest in the supine position for 10 min before the test to ensure cardiovascular stabilization. During this period and throughout the assessment, participants were instructed to remain relaxed, avoid unnecessary movements, and refrain from verbal communication. Ambient noise and external disturbances were minimized [45,46].

The orthostatic test was initiated using the “Orthostatic Test” function available in the Polar Flow system. Following successful heart rate detection, RR intervals were recorded for two minutes in the supine position. Subsequently, an auditory signal instructed participants to stand up, after which RR intervals continued to be recorded for an additional two minutes in the standing position [44,45].

Five parameters were analyzed: resting HR and HRV (supine position), peak HR (highest value recorded immediately after standing), standing HR (mean HR during orthostasis), and standing HRV (HRV recorded during orthostasis).If heart rate acquisition failed, the moisture of the electrodes and positioning of the chest strap were checked and adjusted before a new attempt was performed. Up to five attempts were allowed before exclusion of the recording from analysis [44,45,46].

Following the orthostatic assessment, functional mobility and balance were evaluated using the Timed Up and Go (TUG) test and the Tinetti Performance-Oriented Mobility Assessment, which are widely used clinical instruments for estimating fall risk in older adults.

The TUG test was performed on a flat surface with participants initially seated in a chair with a backrest. Upon a verbal command, participants were instructed to stand up, walk to a cone positioned 3 m away, walk around it, return along the same path, and sit down again. Total execution time was recorded using a stopwatch, beginning at the verbal command and ending when the participant returned to the seated position. Performance was interpreted as follows: <10 s (low risk), 10–19 s (moderate risk), and >20 s (high risk) [26,47].

Balance and gait performance were subsequently assessed using the Tinetti Index, which consists of 16 items distributed across balance and gait domains, with a maximum score of 28 points. The balance domain includes tasks such as rising from a chair, sitting down, standing stability, and response to external perturbation, whereas the gait domain evaluates gait initiation, symmetry, step length, and step height. Scores were classified as follows: 24–28 points (low risk), 19–23 points (moderate risk), and <19 points (high risk) [48].

### 2.7. Bias and Confounding Control

Considering the potential biases inherent to observational studies, strategies were adopted to reduce confounding factors and improve procedural standardization. Evaluators were previously trained, and all assessments were conducted at the same location and during the morning period, ensuring consistent test administration and minimizing variations related to circadian rhythm and environmental conditions.

In addition, self-reported comorbidities were recorded and considered during sample characterization, according to the predefined inclusion and exclusion criteria. To reduce the effect of exercise exposure time as a confounding variable, participants in the active group were selected based on a similar duration of participation in the 60Up program, ranging from 6 to 12 months.

### 2.8. Statistical Analysis

Data were organized, coded, and tabulated in Microsoft Excel 365 (Microsoft Corp., Redmond, WA, USA). Statistical analyses were performed using Jamovi software (The Jamovi Project, Sydney, Australia; version 2.7.34.0).

Continuous variables were expressed as mean ± standard deviation (SD), whereas categorical variables were presented as absolute and relative frequencies.

The distribution of continuous variables was assessed using the Shapiro–Wilk test. Because several variables did not meet normality assumptions and the sample size was relatively small, comparisons between the G60UP Group and the SCG were performed using the Mann–Whitney U test for continuous variables. Categorical variables were compared using Fisher’s exact test.

Associations between falls and clinical-functional variables were investigated using Spearman’s rank correlation coefficient (ρ). Ninety-five percent confidence intervals (95% CI) for correlation coefficients were estimated using bootstrap resampling procedures.

To further explore factors associated with fall occurrence during the previous year (≥1 fall), an exploratory multivariable logistic regression model was performed. Fall occurrence (yes/no) was entered as the dependent variable, whereas age, MMSE score, number of medications, PSQI score, Tinetti score, and participation in the G60UP program were included as independent variables. Results were expressed as odds ratios (OR) with corresponding 95% confidence intervals (95% CI).

Statistical significance was established at *p* < 0.05. All analyses were interpreted considering the exploratory nature of the study, the relatively small sample size, and the cross-sectional design. Given the limited sample size and the number of comparisons performed, the findings should be interpreted cautiously due to the increased possibility of type I error.

## 3. Results

The study included 67 community-dwelling older adults aged 60 to 85 years, comprising 35 participants in the G60UP Group and 32 participants in the Sedentary Comparison Group (SCG). No statistically significant differences were observed between groups regarding age, height, or sex distribution (*p* > 0.05). Women predominated in both groups, representing 86% of the G60UP Group and 78% of the SCG.

Regarding ethnicity, the G60UP Group consisted of 29% Black, 34% White, and 37% mixed-race participants, whereas the SCG included 25% Black, 56% White, and 19% mixed-race participants. Concerning marital status, single or separate individuals accounted for 46% of the G60UP Group and 31% of the SCG, while married or cohabiting participants represented 29% and 44%, respectively. Widowed participants corresponded to approximately one-quarter of both groups.

Educational attainment was similarly distributed between groups. In the G60UP Group, 11% of participants completed higher education, compared with 16% in the SCG. Most participants in both groups had completed elementary or high school education.

Table 1 summarizes the clinical and anthropometric characteristics of the participants. The G60UP group presented significantly higher MMSE scores and lower systolic and diastolic blood pressure values than the group SCG. In addition, participants in the G60 Up group exhibited significantly lower body mass, waist circumference, and WHtR values than those in the SCG.

Despite these between-group differences, no statistically significant differences were observed for BMI or the Conicity Index. Furthermore, both groups presented mean anthropometric values consistent with increased cardiometabolic risk, including elevated waist circumference, WHtR, and BMI values relative to recommended thresholds.

However, these findings should be interpreted with caution. Due to the cross-sectional design, convenience sampling strategy, and potential baseline differences between groups, the observed associations do not imply causality. Residual confounding and selection bias cannot be excluded, as individuals who chose to participate in the G60UP program may have differed from sedentary participants in characteristics such as health status, functional capacity, motivation, and social engagement.

The presence of comorbidities was assessed through self-report using the following question: “Have you ever been diagnosed by a healthcare professional with any of the following health conditions?”, followed by examples including hypertension, diabetes mellitus, dyslipidemia, heart disease, osteoporosis, musculoskeletal disorders, anxiety, depression, and chronic pain.

Most participants reported at least one chronic health condition, and multiple comorbidities were common in both groups. Table 2 presents the distribution of the main self-reported comorbidities.

Regular medication use was assessed through direct questioning. Participants were asked to report all medications used on a daily basis, and medications were subsequently classified according to therapeutic category. Polypharmacy was defined as the regular use of five or more medications.

Table 3 presents the distribution of the most frequently reported medication classes. Compared with the Sedentary Comparison Group (SCG), participants in the G60UP Group reported lower use of analgesic and anti-inflammatory medications and a lower prevalence of polypharmacy. No statistically significant between-group differences were observed for most other medication classes.

The higher prevalence of polypharmacy observed in the SCG may reflect differences in health status and comorbidity burden between groups rather than an independent association with physical activity status.

Polypharmacy was defined as the regular use of five or more medications. Categorical variables were compared using Pearson’s chi-square test or Fisher’s exact test, as appropriate. Continuous variables were compared using Student’s *t*-test or Mann–Whitney U test according to data distribution. Given the exploratory nature of the analyses and the relatively small sample size, results should be interpreted cautiously.

Sleep quality was assessed using the Pittsburgh Sleep Quality Index (PSQI), a validated self-reported questionnaire composed of 19 items covering seven sleep-related domains. Lower scores indicate better perceived sleep quality.

Table 4 presents the PSQI component scores and total PSQI score for both groups. Participants in the G60UP Group reported lower scores in most PSQI domains compared with the Sedentary Comparison Group (SCG), indicating more favorable self-reported sleep quality. Statistically significant differences were observed for subjective sleep quality, sleep latency, sleep duration, sleep disturbances, daytime dysfunction, and total PSQI score. No significant between-group differences were observed for habitual sleep efficiency or use of sleep medication.

Because sleep quality was assessed through self-report, the findings should be interpreted with caution and may be influenced by reporting bias.

Table 5 summarizes the occurrence of falls during the previous 12 months, orthostatic test outcomes, and functional performance measures. Falls were assessed through self-report and categorized as no falls, one fall, or recurrent falls (≥2 falls). A higher proportion of participants in the G60UP Group reported no falls during the previous year, whereas recurrent falls were more frequently reported in the Group SCG. Participants in the G60UP Group also demonstrated higher Tinetti scores and shorter TUG times. No statistically significant differences were observed between groups in heart rate or heart rate variability measures obtained during the orthostatic test.

Because falls were assessed retrospectively through self-report and the study excluded individuals with severe functional impairment, use of walking aids, vestibular disorders, and other conditions affecting balance, these findings should be interpreted cautiously and should not be considered evidence of a causal effect of physical activity on fall occurrence.

Spearman correlation analysis was performed using the entire sample (*n* = 67) to investigate the associations between the number of falls reported during the previous year and clinical-functional indicators. As shown in Table 6, a higher number of falls was significantly associated with older age, greater abdominal adiposity (Conicity Index), poorer sleep quality, greater TUG times, and lower Tinetti scores. The strongest association was observed for Tinetti performance, indicating that poorer balance and gait were related to a greater occurrence of falls. In contrast, no statistically significant associations were observed between falls and MMSE scores or the number of daily medications.

To further explore factors associated with fall occurrence during the previous year (≥1 fall), an exploratory multivariable logistic regression model was performed, including G60UP participation, age, MMSE score, number of medications, PSQI score, and Tinetti score as independent variables (Table 7).

Although participation in the G60UP program was associated with lower odds of reporting a fall (OR = 0.56, 95% CI: 0.13–2.46), this association did not reach statistical significance. Similarly, higher age showed a trend toward increased odds of falls (OR = 1.09, 95% CI: 0.97–1.22, *p* = 0.140), whereas higher Tinetti scores were associated with lower odds of falls (OR = 0.90, 95% CI: 0.76–1.07, *p* = 0.240). MMSE score, number of medications, and PSQI total score were not independently associated with fall occurrence after adjustment for the other variables included in the model.

**Figure 2:**
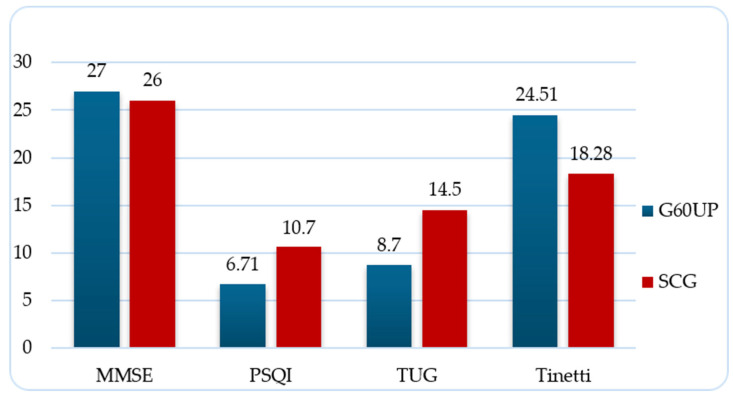
Visual Summary of Cognitive, Sleep-Related, and Functional Outcomes in the G60UP Group and Group SCG. Abbreviations: G60UP, participants of the 60 Up program; SCG, Sedentary Comparison Group; MMSE, Mini-Mental State Examination; PSQI, Pittsburgh Sleep Quality Index; TUG, Timed Up and Go.

Figure 2 Bars represent mean values for MMSE, PSQI, TUG, and Tinetti scores in the G60UP Group and Group SCG. Higher MMSE and Tinetti scores indicate better cognitive and functional performance, whereas lower PSQI and TUG values indicate better sleep quality and mobility, respectively. Detailed statistical comparisons are presented in Table 1, Table 4 and Table 5. Participants in the G60UP Group demonstrated higher MMSE and Tinetti scores, indicating better cognitive performance and functional balance. In addition, the G60UP Group presented lower PSQI scores and shorter TUG times, reflecting better sleep quality and mobility performance, supporting the hypothesis that regular participation in community-based exercise programs may contribute to improved cognitive, sleep-related, and functional health outcomes in older adults. 

**Figure 3:**
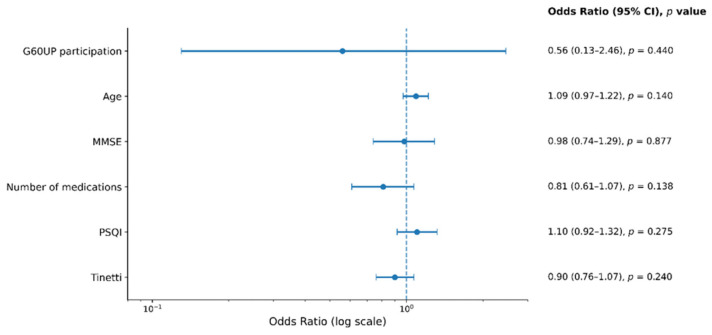
Forest plot of the exploratory multivariable logistic regression model for fall occurrence during the previous year (≥1 fall).

Figure 3 presents the results of the exploratory multivariable logistic regression model evaluating factors associated with the occurrence of at least one fall during the previous year. After adjustment for the variables included in the model, none of the predictors reached statistical significance (all *p* > 0.05). Participation in the G60UP program was associated with a lower likelihood of reporting a fall (OR = 0.56), suggesting a potential protective effect; however, the confidence interval was wide and crossed the null value (95% CI: 0.13–2.46). Similarly, higher Tinetti scores tended to be associated with a lower probability of falls (OR = 0.90), whereas increasing age (OR = 1.09), higher PSQI scores (OR = 1.10), and a greater number of medications (OR = 0.81) showed no independent associations with fall occurrence. Overall, the findings indicate that although several variables demonstrated clinically relevant trends, the model did not identify independent predictors of falls, likely due to the limited sample size and the relatively small number of fall events, which reduced statistical power.

## 4. Discussion

### 4.1. Cardiometabolic Profile and Abdominal Adiposity

The clinical and anthropometric results presented in Table 1 demonstrated that participants enrolled in the G60UP program exhibited a more favorable cardiovascular and anthropometric profile than those in the Sedentary Comparison Group (SCG). Although no significant differences were observed for age, BMI, Conicity Index, visceral fat level, or resting heart rate, the G60UP group presented significantly lower systolic and diastolic blood pressure values. According to the 2025 Brazilian Guidelines for Arterial Hypertension, blood pressure values similar to those observed in the SCG are associated with increased cardiovascular risk, whereas lower values, such as those observed in the G60UP group, are generally considered more favorable for cardiovascular health [50]. Resting heart rate values in both groups remained within the expected physiological range for older adults, consistent with reference values reported by Sammito and Böckelmann [49]. 

Participants in the SCG also presented significantly higher body mass, waist circumference, and waist-to-height ratio values. Although BMI did not differ significantly between groups, both groups exhibited mean BMI values compatible with overweight status, while waist circumference and WHtR exceeded recommended thresholds, indicating increased cardiometabolic risk. Previous studies have shown that indicators of abdominal adiposity, particularly waist circumference and central fat distribution, are strongly associated with cardiometabolic risk in older adults and may provide more clinically relevant information than BMI alone [51,52]. Likewise, the higher mean visceral fat level observed in the SCG is consistent with reference values proposed for bioelectrical impedance analysis, which associate elevated visceral fat with less favorable metabolic and cardiovascular profiles [40]. 

The Conicity Index demonstrated a weak-to-moderate positive correlation with fall occurrence (ρ = 0.281; *p* = 0.021), suggesting that greater abdominal adiposity was associated with a higher frequency of falls. Similarly, participants in the Sedentary Comparison Group presented higher waist circumference, waist-to-height ratio, and visceral fat values, reinforcing the association between central adiposity and a less favorable cardiometabolic profile. Previous studies have shown that excess abdominal adiposity may negatively affect muscle function, mobility, and postural control, thereby increasing fall vulnerability among older adults [52]. Taken together, these findings suggest that central adiposity indicators, including waist circumference, waist-to-height ratio, visceral fat level, and the Conicity Index, may represent useful markers of both cardiometabolic health and fall-related vulnerability. However, because of the observational cross-sectional design, these associations should be interpreted cautiously and do not establish causal relationships.

### 4.2. Multimorbidity and Chronic Diseases

The SCG reported a higher prevalence of multimorbidity and greater medication use than participants enrolled in the G60UP program (Table 2). Hypertension and diabetes mellitus were among the most frequently reported chronic conditions in both groups, although their prevalence tended to be lower in the G60UP group. Similarly, participants enrolled in the 60 Up program presented significantly lower systolic and diastolic blood pressure values, findings that are consistent with previous studies reporting associations between regular physical activity and more favorable cardiovascular profiles [53,54,55,56]. 

However, not statistically significant between-group differences were observed for hypertension, diabetes mellitus, dyslipidemia, heart disease, osteoporosis, the absence of reported diseases, or the total number of reported comorbidities. Because all clinical conditions were self-reported and participants were not randomly allocated, these findings should be interpreted with caution. Residual confounding, recall bias, and selection bias cannot be excluded, as healthier and more functionally independent older adults may have been more likely to participate in the community-based exercise program.

### 4.3. Mental Health and Musculoskeletal Conditions

Anxiety and depression were reported by 31% of participants in the G60UP group and 59% of those in the SCG, although most participants did not report using specific medications for these conditions. Previous studies have shown that mental health disorders are frequently underdiagnosed and undertreated among older adults [57]. Musculoskeletal disorders and chronic pain were also reported less frequently by participants enrolled in the G60UP program.

These findings are consistent with studies suggesting that physically active older adults generally report better physical and psychological health. Nevertheless, because this was an observational cross-sectional study, it cannot be determined whether participation in the exercise program contributed to these differences or whether individuals with better baseline physical and mental health were more likely to participate and remain engaged in the program. Therefore, the observed differences should be interpreted as associations rather than evidence of a beneficial effect of the program.

### 4.4. Polypharmacy and Fall Risk

Polypharmacy, defined as the concomitant use of five or more medications, is common among older adults and has been associated with adverse clinical outcomes, drug interactions, and increased fall risk [58]. Antihypertensive medications were the most frequently used drugs in both groups, whereas the sedentary comparison group reported higher consumption of analgesics and anti-inflammatory drugs, reflecting a greater burden of chronic pain (Table 3).

Daily medication use showed a positive correlation with fall occurrence (*r* = 0.24), reinforcing polypharmacy as a factor associated with falls in this sample (Table 6). Although participants in the 60 Up group reported lower medication use, the cross-sectional design does not allow determination of whether participation in the program influenced medication consumption or whether healthier individuals were more likely to engage in regular physical activity.

### 4.5. Functional Performance, Mobility, and Cognition

Moderate correlations were observed between falls and functional performance assessed by the Tinetti Index and the TUG test (Table 6), indicating that poorer balance and mobility were associated with greater fall occurrence. Table 6 also demonstrated a negative correlation between MMSE scores and falls, suggesting that lower cognitive performance was associated with increased fall occurrence.

Participants enrolled in the 60 Up program demonstrated better functional and cognitive performance than those in the sedentary comparison group. Similar findings have been reported by Tarazona-Santabalbina et al. (2016), who observed better functional and cognitive outcomes among older adults participating in supervised exercise programs [32]. Likewise, Gené Huguet et al. (2022) reported that older adults involved in multifactorial health interventions presented lower levels of frailty and reduced healthcare utilization over long-term follow-up, highlighting the potential relationship between functional capacity and healthy aging [59]. 

The relationship between cognition and functional performance has been previously described in the literature. Castilho et al. (2018) demonstrated that cognitive status influences postural balance in older adults, suggesting that cognitive decline may contribute to impaired motor control and greater instability during daily activities [60]. Racey et al. (2021) also highlighted that cognitive and functional deficits are associated with impaired gait and balance, increasing dependence, institutionalization, and mortality [16]. Similarly, Zhang et al. (2023) reported associations between cognitive impairment, depressive symptoms, reduced muscle strength, and increased fall occurrence [61]. 

Evidence regarding the effects of physical activity on cognition remains heterogeneous. Sanders et al. (2020), in a randomized controlled trial involving older adults with dementia, observed improvements in some physical performance measures but limited effects on cognitive outcomes, suggesting that the relationship between exercise and cognition may depend on factors such as exercise intensity, participant characteristics, and duration of follow-up [62]. In addition, Sousa et al. (2023) reported better quality of life among physically active older women compared with sedentary peers, reinforcing the association between active lifestyles and more favorable health indicators [36]. 

Taken together, the present findings reinforce the association between functional performance, cognitive status, and fall occurrence in older adults. Nevertheless, given the cross-sectional design and exploratory nature of the analyses, causal relationships cannot be established, and residual confounding factors cannot be excluded.

### 4.6. Sleep Quality, Polypharmacy and Falls

Recent evidence suggests that the interaction between polypharmacy and poor sleep quality may increase fall risk, particularly among individuals using antihypertensive, antidepressant, or sedative medications [63,64]. Poor sleep quality may contribute to daytime sleepiness, impaired attention, and reduced recovery capacity, factors that have been associated with falls.

In the present study, participants in the SCG showed poorer sleep quality and a higher prevalence of polypharmacy, both of which were associated with greater fall occurrence (Table 5 and Table 6). These results are consistent with previous studies reporting links between insomnia, multimorbidity, polypharmacy, and falls [37,38]. 

An additional aspect to consider is that fall occurrence is influenced by multiple interrelated factors beyond chronological aging alone. Stolt et al. (2020) reported a high prevalence of accidental falls among middle-aged women and identified associations with musculoskeletal pain, poor sleep quality, excessive daytime sleepiness, depressive symptoms, and metabolic syndrome [65]. These findings suggest that sleep disturbances may coexist with other clinical and behavioral factors that contribute to fall vulnerability. In the present study, poorer sleep quality was associated with greater fall occurrence, reinforcing the multifactorial nature of falls and highlighting the importance of considering sleep health within a broader clinical context.

Participants enrolled in the 60 Up program reported better sleep quality and fewer falls than those in the sedentary comparison group. However, these differences should not be interpreted as evidence that participation in the program directly improved sleep quality or reduced falls. Although previous intervention studies have reported improvements in sleep parameters following structured exercise programs [34,39]. the present study can only demonstrate that participation in the 60 Up program was associated with more favorable sleep-related outcomes. Longitudinal and randomized studies are required to determine whether these associations reflect causal effects.

### 4.7. Sleep, Cognition, and Falls: Implications for Healthy Aging

These findings reinforce the multidimensional nature of falls in older adults, demonstrating that poorer sleep quality and lower cognitive performance were associated with a higher incidence of falls in the overall sample. Although these associations did not remain statistically significant in the exploratory multivariate logistic regression model, they are consistent with previous evidence suggesting that sleep and cognition play complementary roles in functional performance and vulnerability to falls [16,19,36]. 

Poor sleep quality can negatively influence attention, executive function, reaction time, and postural control, thereby increasing susceptibility to falls. Conversely, cognitive impairment can impair gait planning, environmental perception, and the ability to perform dual-task activities—aspects essential for safe mobility in older adults [16,19,36]. These mechanisms likely interact with one another rather than acting independently, reinforcing the concept that falls result from multiple interrelated physiological and behavioral factors.

Participants enrolled in the G60UP program showed better scores on both the MMSE and the PSQI compared to members of the sedentary group. However, due to the cross-sectional design, these findings should be interpreted as associations rather than evidence that program participation improved cognition or sleep quality. Healthier, socially engaged, and functionally independent older adults may have been more likely to participate in the community-based exercise program, potentially contributing to the observed differences.

From a public health perspective, these findings highlight the importance of incorporating multidimensional screening into fall prevention strategies. In addition to balance and mobility assessments, evaluating sleep quality and cognitive performance can help identify older adults at higher risk of falls and guide individualized preventive interventions.

### 4.8. Orthostatic Test, Autonomic Function, and Physical Activity

The orthostatic test was included as a complementary assessment of autonomic cardiovascular responses during postural transition. Previous studies have suggested that regular physical activity may be associated with favorable autonomic adaptations, including changes in heart rate variability (HRV) and cardiovascular regulation [66,67,68]. 

However, in the present study, not statistically significant between-group differences were observed for resting HR, resting HRV, peak orthostatic response, or standing HRV (Table 5; *p* > 0.05). Both groups presented values within expected reference ranges. In contrast, significant differences were observed for systolic blood pressure (SBP) and diastolic blood pressure (DBP), indicating differences in hemodynamic profile between participants enrolled in the 60 Up program and the Group SCG.

Although standing HRV showed a numerical trend toward group differences, the absence of statistical significance precludes firm conclusions regarding autonomic function. Furthermore, HRV parameters were not significantly associated with fall occurrence in the present sample.

Previous studies have demonstrated that the orthostatic test is a useful tool for evaluating autonomic cardiovascular adaptation during postural transitions in older adults. Pletsch et al. (2018) reported alterations in HRV indices during orthostatic challenge in hypertensive older adults, suggesting that aging and cardiovascular conditions may influence autonomic modulation [46]. Likewise, Gerritsen and Band (2018) emphasized the importance of vagal regulation and autonomic flexibility for cardiovascular adaptation and overall physiological resilience [45]. In the present study, despite the absence of significant between-group differences in HRV parameters, both groups demonstrated physiological responses compatible with postural adaptation, remaining within expected reference ranges.

The absence of significant HRV differences may reflect the influence of multiple factors, including age, comorbidities, medication use, and substantial interindividual variability. Borges et al. (2021) highlighted that postural control depends on the interaction of sensory, neuromuscular, and physiological regulatory systems, suggesting that balance and mobility outcomes may not be fully explained by isolated autonomic measures [69]. Therefore, although participants enrolled in the 60 Up program presented better functional performance and lower blood pressure values, the present findings do not support the conclusion that participation in the program was associated with superior autonomic regulation.

### 4.9. Balance, Mobility, and Fall Prevention

Postural balance depends on coordinated neuromuscular responses that may be affected by aging and reduced physical activity, contributing to mobility limitations and increased fall occurrence [66]. In the present study, participants enrolled in the 60 Up program presented higher Tinetti scores and shorter TUG execution times than those in the sedentary comparison group, indicating better balance and functional mobility.

Correlation analyses demonstrated moderate associations between falls and both Tinetti scores (ρ = −0.416) and TUG performance (ρ = 0.321), suggesting that poorer balance and mobility were associated with greater fall occurrence. These findings are consistent with previous studies reporting associations between impaired functional performance and falls in older adults [70,71].

Participants in the SCG also demonstrated longer TUG execution times and greater variability in performance, suggesting lower functional mobility and greater heterogeneity within the group. Previous studies have reported that TUG times below 10 s are generally associated with preserved functional mobility, whereas values above 13.5 s may indicate increased fall risk [72,73]. The present findings are broadly consistent with this literature.

The observed differences in functional performance may also be interpreted in light of the broader relationship between cardiovascular health and mobility in older adults. Tavares et al. (2024) reported significant associations between cardiovascular risk factors and Timed Up and Go performance among participants of public physical activity programs, suggesting that poorer cardiometabolic profiles may be accompanied by reduced functional mobility and increased vulnerability to adverse outcomes [74]. In the present study, participants in the sedentary comparison group exhibited higher blood pressure levels, greater abdominal adiposity, and longer TUG execution times, reinforcing the coexistence of functional and cardiometabolic risk factors frequently observed in aging populations.

In addition, Laborde et al. (2017) and Singh et al. (2018) emphasized that heart rate variability reflects the dynamic interaction between autonomic control and physiological adaptation, although its interpretation should consider multiple clinical and behavioral factors [44,75]. Furthermore, the use of beta-blockers, which was common among participants due to the high prevalence of hypertension, may influence cardiovascular responses during physical tasks and autonomic measurements [44,75]. Although no significant between-group differences were observed for HRV variables in the present study, these factors may contribute to interindividual variability in functional performance and should be considered when interpreting mobility and fall-related outcomes in older adults.

Previous intervention studies have demonstrated improvements in balance and mobility following structured exercise programs [70,71]. However, because the present study used a cross-sectional observational design, the differences observed between groups should be interpreted as associations rather than evidence that participation in the 60 Up program caused better functional performance or reduced fall occurrence.

Importantly, in the exploratory multivariable logistic regression model, neither Tinetti score nor participation in the 60 Up program remained independently associated with fall occurrence after adjustment for age, MMSE score, medication use, and sleep quality. These findings suggest that the observed associations may be influenced by multiple interrelated factors and should therefore be interpreted with caution.

### 4.10. Clinical Implication

The present findings suggest that poorer balance and mobility, greater abdominal adiposity, poorer sleep quality, and advanced age were associated with a higher incidence of falls among community-dwelling older adults. These results highlight the importance of a multidimensional assessment in clinical practice, including the analysis of functional performance, anthropometric indicators, sleep quality, medication use, and cognitive status.

Participants enrolled in the “60 Up” program generally exhibited more favorable functional, cognitive, and sleep profiles than those in the sedentary comparison group. However, due to the observational and cross-sectional nature of the study, these differences cannot be directly attributed to participation in the exercise program. Alternative explanations, including selection bias and differences in baseline health status, motivation, and social engagement, cannot be ruled out.

Therefore, the present findings should be interpreted as associations that may contribute to identifying factors related to falls and healthy aging, rather than as evidence of causal effects.

### 4.11. Limitations and Future Perspectives

Certain limitations should be considered when interpreting the presented results. First, the occurrence of falls was assessed via self-report, which may have introduced recall bias. Second, the relatively small sample size and the predominance of female participants may limit the generalizability of the results.

Furthermore, given the exploratory nature of the analyses and the cross-sectional design, it is not possible to infer causality or determine whether the observed differences preceded or resulted from participation in the exercise program. Selection bias is also possible, as individuals who choose to participate in community exercise programs may differ from sedentary individuals in terms of health status, functional capacity, motivation, and social engagement. Consequently, the results presented should be considered hypothesis-generating rather than confirmatory. Future longitudinal studies with larger, more representative samples are needed to clarify the temporal relationships between physical activity, functional performance, cognition, sleep quality, and falls. Prospective and intervention studies would be particularly valuable for determining whether participation in community-based exercise programs influences fall-related outcomes over time.

## 5. Conclusions

The results of this study contribute to identifying factors potentially associated with falls in older adults and highlight the importance of multidimensional assessment, including functional, cognitive, anthropometric, and sleep-related characteristics. In this sample of community-dwelling older adults, the occurrence of falls was associated with poorer balance and mobility, greater abdominal adiposity, poorer sleep quality, and advanced age. Participants in the “60 Up” program reported fewer falls and exhibited more favorable functional, cognitive, anthropometric, and sleep-related profiles than those in the sedentary comparison group.

However, due to the cross-sectional observational design, these findings should be interpreted as associations rather than evidence of causal relationships. Additional longitudinal and intervention studies are needed to clarify the temporal relationships between these variables and determine whether participation in public physical activity programs influences fall-related outcomes over time.

## Figures and Tables

**Table 1:** Clinical and anthropometric characteristics of the G60UP group and group SCG.

Variable	G60UP Mean ± SD	SCG Mean ± SD	*p*-Value
Age (years)	68.3 ± 4.3	70.4 ± 7.0	0.248
MMSE (score)	27.0 ± 2.3	25.9 ± 2.8	0.023
Systolic BP (mmHg)	128 ± 9	140 ± 20	0.002
Diastolic BP (mmHg)	76 ± 9	82 ± 9	0.026
Resting HR (bpm)	70.6 ± 8.9	67.6 ± 6.8	0.190
Body Mass (kg)	68.0 ± 13.0	78.0 ± 22.0	0.029
Height (cm)	158.71 ± 9.80	160.56 ± 10.22	0.497
BMI (kg/m^2^)	27.0 ± 4.0	29.0 ± 6.0	0.098
Waist Circumference (cm)	93 ± 12	103 ± 19	0.011
WHtR (cm)	0.58 ± 0.07	0.65 ± 0.12	0.012
Conicity Index (cm, kg)	1.30 ± 0.12	1.36 ± 0.18	0.072
Visceral Fat Level	9.5 ± 3.6	12.0 ± 5.0	0.204

Abbreviations: G60UP, participants of the 60 Up program; SCG, Sedentary Comparison Group; MMSE, Mini-Mental State Examination; BP, Blood Pressure; HR, Heart Rate; BMI, Body Mass Index; WHtR, Waist-to-Height Ratio; SD, Standard Deviation. Continuous variables are presented as mean ± standard deviation (SD). Normality was assessed using the Shapiro–Wilk test. Because several variables did not satisfy normality assumptions and the sample size was relatively small, between-group comparisons were performed using the Mann–Whitney U test. Statistical significance was set at *p* < 0.05.

**Table 2:** Self-reported comorbidities in the G60UP group and group SCG.

Variables	G60UP *n* (%)	SCG *n* (%)	*p*-Value
Hypertension	24 (69%)	24 (75%)	0.4283
Diabetes mellitus	13 (37%)	11 (34%)	0.0980
Dyslipidemia	16 (46%)	13 (41%)	0.6801
Heart disease	6 (17%)	8 (25%)	0.4371
Mental disorders (anxiety/depression)	11 (31%)	19 (59%)	0.0214
Osteoporosis	4 (11%)	7 (22%)	0.2556
Musculoskeletal disorders	12 (34%)	26 (81%)	<0.0001
Chronic pain (>3 months)	11 (31%)	29 (91%)	<0.0001
No reported diseases	3 (9%)	1 (3%)	0.3548
Number of reported comorbidities (Mean ± SD)	1.40 ± 0.83	1.68 ± 0.74	0.3363

Abbreviations: G60UP, participants of the 60 Up program; SCG, Sedentary Comparison Group; SD, standard deviation. Comorbidities were self-reported and were not verified through medical records. Continuous variables are presented as mean ± standard deviation (SD) and were compared between groups using the Mann–Whitney U test. Categorical variables are presented as absolute and relative frequencies and were compared using Fisher’s exact test. Statistical significance was set at *p* < 0.05.

**Table 3:** Medication use in the G60UP group and group SCG.

Variable	G60UP *n* (%)	SCG *n* (%)	*p*-Value
Antihypertensive/antiarrhythmic drugs	25 (71%)	26 (81%)	0.400
Diuretics	8 (23%)	11 (34%)	0.416
Antihypertensive Beta-blockers	5 (14%)	10 (31%)	0.143
Antidiabetic drugs	12 (34%)	11 (34%)	1.000
Antiulcer drugs	5 (14%)	8 (25%)	0.358
Lipid-lowering agents	11 (31%)	7 (22%)	0.420
Antidepressants/anxiolytics	6 (17%)	8 (25%)	0.551
Analgesics/anti-inflammatory drugs	2 (6%)	16 (50%)	<0.001
Polypharmacy (≥5 medications)	4 (100%)	15 (47%)	0.106
4 medications	8 (100%)	4 (12%)	<0.001
0–3 medications	23 (100%)	13 (41%)	<0.001
Number of medications (Mean ± SD)	2.86 ± 1.57	4.41 ± 3.04	0.036

Abbreviations: G60UP, participants of the 60 Up program; SCG, Sedentary Comparison Group; SD, standard deviation. Note: Comorbidities and medication use were self-reported and were not confirmed through medical records. Categorical variables were compared using Fisher’s exact test. Continuous variables are presented as mean ± standard deviation (SD). Continuous variables were compared using the Mann–Whitney U test, according to the data distribution.

**Table 4:** Pittsburgh Sleep Quality Index (PSQI) outcomes in the G60UP group and group SCG.

PSQI Domain	G60UP (Mean ± SD)	SCG (Mean ± SD)	*p*-Value
Subjective sleep quality	0.94 ± 0.68	1.78 ± 0.78	<0.001
Sleep latency	1.43 ± 0.88	2.19 ± 0.92	<0.001
Sleep duration	0.86 ± 1.00	1.66 ± 0.80	<0.001
Habitual sleep efficiency	1.00 ± 1.14	1.36 ± 1.08	0.131
Sleep disturbances	1.11 ± 0.68	1.59 ± 0.64	0.008
Use of sleep medication	0.40 ± 0.98	0.63 ± 0.92	0.031
Daytime dysfunction	0.94 ± 1.47	1.56 ± 0.93	0.004
Total PSQI score	6.71 ± 3.06	10.70 ± 3.83	<0.001

Abbreviations: G60UP, participants of the 60 Up program; SCG, Sedentary Comparison Group; PSQI, Pittsburgh Sleep Quality Index; SD, standard deviation. Note: PSQI scores were obtained through self-report. Continuous variables are presented as mean ± standard deviation (SD). Comparisons between groups were performed using the Mann–Whitney U test. Higher PSQI scores indicate poorer sleep quality. Total PSQI scores should be interpreted as follows: 0–4 = good sleep quality; 5–10 = poor sleep quality; >10 = severe sleep impairment [43].

**Table 5:** Falls, orthostatic test outcomes, and functional performance in the G60UP group and Sedentary Comparison Group (SCG).

Variable	G60UP	SCG	*p*-Value
Falls during the previous year	G60UP *n* (%)	SCG *n* (%)	-
No falls	27 (77%)	15 (47%)	0.013
1 fall	7 (20%)	9 (28%)	0.568
≥2 falls	1 (3%)	8 (25%)	0.011
Orthostatic and functional variables	G60UP (Mean ± SD)	SCG (Mean ± SD)	-
Mean number of falls	0.26 ± 0.51	0.78 ± 0.83	0.005
Resting HR (bpm)	70.25 ± 9.97	67.94 ± 7.69	0.462
Resting HRV (ms)	21.41 ± 13.48	23.78 ± 19.17	0.559
Peak HR (bpm)	83.09 ± 18.67	82.16 ± 14.10	0.282
Standing HR (bpm)	78.59 ± 10.70	76.19 ± 9.83	0.950
Standing HRV (ms)	17.90 ± 15.47	24.05 ± 25.99	0.277
Tinetti Balance Score	13.49 ± 1.80	9.53 ± 3.03	<0.001
Tinetti Gait Score	11.03 ± 1.62	8.66 ± 3.98	0.004
Tinetti Score	24.51 ± 2.54	18.28 ± 5.94	<0.001
TUG (s)	8.66 ± 1.63	13.00 ± 4.31	<0.001

Abbreviations: G60UP, participants of the 60 Up program; SCG, Sedentary Comparison Group; HR, heart rate; HRV, heart rate variability; ms, milliseconds; SD, standard deviation; TUG, Timed Up and Go. Note: Falls were assessed by self-report for the previous 12 months and may be subject to recall bias. Categorical variables were compared using Fisher’s exact test. Continuous variables were compared using the Mann–Whitney U test. Interpretation of selected measures: Resting heart rate values between 60 and 100 bpm are generally considered within the normal range [49]. Tinetti scores range from 0 to 28 points, with 24–28 indicating low fall risk, 19–23 moderate fall risk, and <19 high fall risk [48]. For the TUG, values ≤ 10 s generally indicate preserved functional mobility, whereas longer TUG times indicate poorer functional mobility [26].

**Table 6:** Spearman correlations between fall occurrence during the previous year and clinical, anthropometric, and functional variables in the total sample (*n* = 67).

Variable	Spearman’s ρ	95% CI	*p*-Value
MMSE	−0.199	−0.423 to 0.053	0.107
Daily medication use	−0.004	−0.281 to 0.266	0.973
Conicity Index	0.281	0.019 to 0.503	0.021
Tinetti Score	−0.416	−0.622 to −0.166	<0.001
TUG(s)	0.321	0.084 to 0.527	0.008
PSQI Score	0.243	−0.012 to 0.479	0.047
Age	0.328	0.090 to 0.536	0.007

Abbreviations: MMSE, Mini-Mental State Examination; TUG, Timed Up and Go; PSQI, Pittsburgh Sleep Quality Index. Correlations were calculated using Spearman’s rank correlation coefficient (ρ) due to the ordinal nature of the fall variable and the non-normal distribution of several variables. Confidence intervals (95% CI) are reported for correlation coefficients. Statistical significance was set at *p* < 0.05. Given the exploratory nature of the study, the relatively small sample size, and the multiple correlations performed, the findings should be interpreted with caution and considered hypothesis-generating. Source: Research data.

**Table 7:** Exploratory Multivariable logistic regression model for fall occurrence during the previous year (≥1 fall) in the total sample (*n* = 67).

Variable	Odds Ratio (OR)	95% CI	*p*-Value
G60UP participation	0.44	0.10–1.96	0.282
Age	1.11	1.00–1.25	0.060
MMSE	0.96	0.73–1.25	0.745
Number of medications	0.85	0.63–1.15	0.295
Tinetti Score	0.90	0.76–1.07	0.240
PSQI Score	1.09	0.91–1.31	0.326

Abbreviations: OR Odds Ratio; CI, Confidence Interval; MMSE, Mini-Mental State Examination; PSQI, Pittsburgh Sleep Quality Index. An exploratory multivariable logistic regression model was performed with fall occurrence during the previous year (≥1 fall) as the dependent variable. Independent variables included participation in the 60 Up program, age, MMSE score, number of medications, Tinetti score, and PSQI score. Results are presented as odds ratios (OR) with 95% confidence intervals (95% CI). Statistical significance was set at *p* < 0.05. Given the relatively small sample size and the limited number of fall events, the model should be considered exploratory and interpreted with caution. Source: Research data.

## Data Availability

The raw data supporting the conclusions of this article will be made available by the authors on request.
